# Cyclophosphamide for the treatment of acute lymphoblastic leukemia: A protocol for systematic review

**DOI:** 10.1097/MD.0000000000014293

**Published:** 2019-02-01

**Authors:** Yue-rong Zhao, Hong-mei Song, Lei Ni

**Affiliations:** a Department of Hematology; b Department of Infectious Diseases, First Affiliated Hospital of Jiamusi University, Jiamusi, 154002, China.

**Keywords:** acute lymphoblastic leukemia, cyclophosphamide, efficacy, safety, systematic review

## Abstract

**Background:**

Previous clinical trials have reported that cyclophosphamide can be used for the treatment of acute lymphoblastic leukemia (ALL). However, its efficacy is still unclear. In this systematic review study, we aim to evaluate its efficacy and safety for ALL.

**Methods:**

The following 9 databases will be searched from their inception to the present: Cochrane Central Register of Controlled Trials (CENTRAL), EMBASE, MEDLINE, Cumulative Index to Nursing and Allied Health Literature (CINAHL), Allied and Complementary Medicine Database (AMED), and four Chinese databases. The randomized controlled trials or case control studies of cyclophosphamide that assess the clinical efficacy and safety in patients with ALL are included. The methodological quality of all eligible included studies will be assessed by the Cochrane risk of bias tool.

The primary outcome measurement will be all-cause mortality at the period of treatment and follow-up. The secondary outcome measurements will include the health-related quality of life (HRQL), postinduction complete remission (CR) rate, event-free survival (EFS), relapse rate, and adverse events. Two authors will independently select eligible studies, exact data, and assess the methodological quality of included studies. RevMan 5.3 software will be used to synthesize the data. Reporting bias will be evaluated by the funnel plots, Begg, and Egger tests.

**Results:**

This systematic review will evaluate the clinical efficacy and safety of cyclophosphamide for ALL.

**Dissemination and ethics:**

The findings of this review will summarize the present evidence of cyclophosphamide for ALL, and may provide guidance for clinical practice of cyclophosphamide for ALL. Its results will be published through peer-reviewed journals. This study does not need ethic approval, because it will not involve the individual data.

**Systematic review registration:**

PROSPERO CRD42018119333.

## 1 Introduction

Acute lymphoblastic leukemia (ALL) is the most common hematologic malignancy cancer of bone marrow.^[1–3]^ It is featured by the overproduction of immature lymphoblasts.^[4–7]^ It has been reported that about 6000 patients are diagnosed with this condition annually, and it accounts more than 75% acute leukemias in children, and 20% of all leukemias in adults.^[8–11]^ Although several treatments are available for such disorder, its relapse rate is still about 15%–20% in ALL. Furthermore, the cue rate is much lower after the relapse.^[12]^

Despite the prevalence and incidence of ALL is very high, its treatments are still suffered from limited efficacy and poorly supported.^[13–19]^ Cyclophosphamide has been reported to improve the efficacy of patients with ALL and has achieved promising efficacy.^[13–28]^ However, no systematic review has further evaluated its efficacy of higher level evidence with more convinced conclusion. Thus, it is very necessary to conduct a systematic review and meta-analysis to assess the efficacy and safety of cyclophosphamide for the treatment of ALL.

## 2 Methods

### 2.1 Objective

This systematic review and meta-analysis aims to assess the efficacy and safety of cyclophosphamide for the treatment of ALL.

### 2.2 Study registration

This protocol was designed according to the Preferred Reporting Items for Systematic Reviews and Meta-Analysis Protocol (PRISMA-P) statement guidelines,^[29]^ and it has been registered in PROSPERO with CRD42018119333.

### 2.3 Inclusion criteria for study selection

#### 2.3.1 Study type

This review will consider the randomized controlled trials (RCTs) or case control studies that assessed the efficacy and safety of cyclophosphamide on ALL without any restrictions, such as age, race, and region. Any other studies including animal studies, case reports, case series, observational studies, qualitative studies, letters, comments, and reviews will all be excluded.

#### 2.3.2 Participant type

Patients with ALL, regarding gender, and age will be included.

#### 2.3.3 Intervention type

Intervention of any type of cyclophosphamide treatment will be included. However, the combination of cyclophosphamide with other treatments will be excluded. Control therapy will include placebo, or other interventions, except the cyclophosphamide will be considered.

#### 2.3.4 Outcome measurement type

The primary outcome includes all-cause mortality at the period of treatment and follow-up. The secondary outcomes consist of health-related quality of life (HRQL), postinduction complete remission (CR) rate, event-free survival (EFS), relapse rate, and adverse events.

### 2.4 Search methods for the identification of studies

#### 2.4.1 Electronic searches

The following databases will be searched from the inception to the present: Cochrane Central Register of Controlled Trials (CENTRAL), EMBASE, MEDLINE, the Cumulative Index to Nursing and Allied Health Literature (CINAHL), the Allied and Complementary Medicine Database (AMED), and four Chinese databases of Chinese Biomedical Literature Database (CBM), China National Knowledge Infrastructure (CNKI), VIP Information (VIP), and Wanfang Data (WANFANG). The details of search strategy for CENTRAL are shown in Table 1. Similar strategies will be used and applied for all other electronic databases.

**Table 1:**
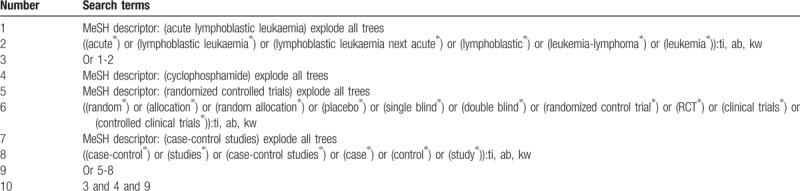
Search strategy applied in CENTRAL database.

#### 2.4.2 Search for other resources

The ongoing and recently completed studies will also be searched from the clinical registration website. Additionally, the reference lists of potentially related studies will also be considered to search in order to avoiding missing any other potential eligible studies.

### 2.5 Data collection and analysis

#### 2.5.1 Study selection

**Figure 1:**
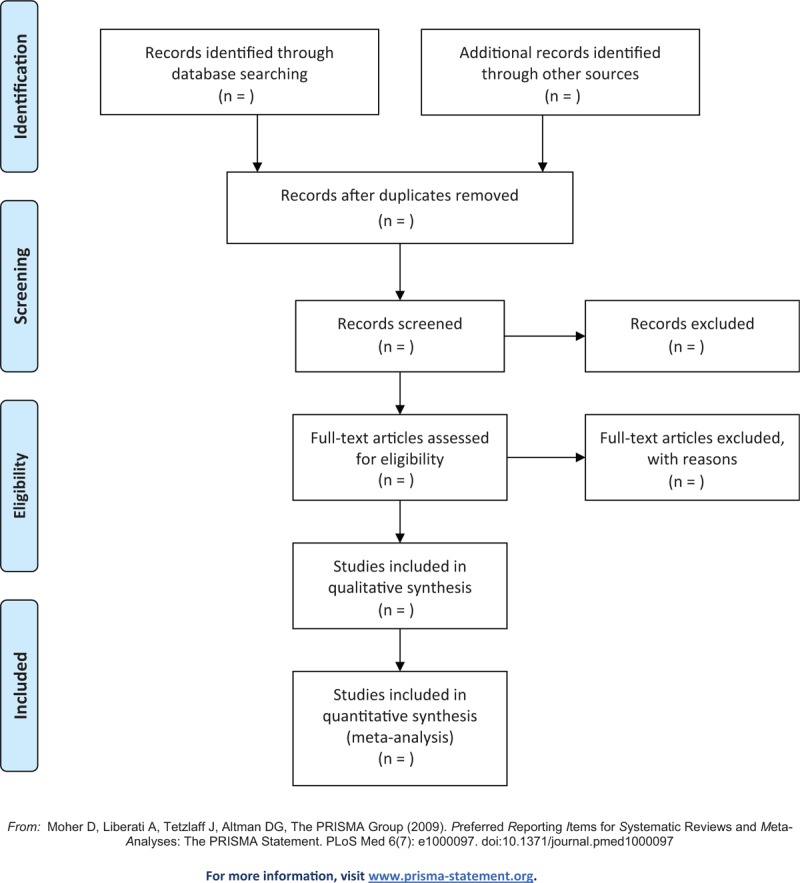
Flow diagram of study selection process.

Two authors will independently select the initial titles and abstracts. Then, the full text of potential related studies will be further reviewed for inclusion according to the study eligibility. All selection procedures will be applied according to the PRISMA flow chart. A third author will resolve the disagreements by discussion if there will be occurred between the two authors. The flowchart of study selection is presented in Figure 1.

#### 2.5.2 Data extraction and management

Two authors will independently extract data from the included studies using the redefined standard data extraction form. The exacted information includes author, published year, region, age, gender, ethnicity, disease type, treatment dosage, outcome measurements, and so on. If any disagreement regarding the data extraction between these 2 authors occurred, the matter will be resolved by a third author through discussion.

#### 2.5.3 Risk of bias in included studies

Two authors will also independently assess the risk of bias in each included study by using the Cochrane Handbook for Systematic Reviews of Interventions Tool. The evaluation includes following 7 specific aspects according to the Cochrane Handbook for bias risk assessment. Any divisions will also be solved by a third author involved.

#### 2.5.4 Measurement of treatment effect

We will use mean difference (MD) or standardized mean difference (SMD) with 95% confidence intervals (CIs) to analyze the continuous data. As for dichotomous data, we will use risk ratio (RR) to perform the treatment effect with 95% CIs.

#### 2.5.5 Unit of analysis

We will evaluate the data of first study period in cross-over studies in order to avoid the carryover effects. If included studies have multiple intervention groups, all related treatment and control groups will be combined into a single unit to further avoid the unit analysis.

#### 2.5.6 Dealing with missing data

If the data information is missing, or insufficient or unclear, the original authors will be contact to request these data. If the missing data are not obtainable, the available data will be analyzed and will be discussed in the discussion as limitations.

#### 2.5.7 Subgroup analysis

Subgroup analysis will be performed for different treatment forms if the substantial heterogeneity will be identified, such as different treatment types, control therapies, and outcome measurements.

#### 2.5.8 Assessment of heterogeneity and data synthesis

Heterogeneity will be assessed by the *I*^2^ and *χ*^2^ tests in this study. When the value of I^2^ was less than 50%, no statistical heterogeneity will be considered, and fixed-effect model will be utilized. Otherwise, substantial heterogeneity will be considered and random-effect model will be used to analyze the outcome data. In such situation, subgroup analysis will be conducted and potential reasons will be analyzed to investigate the potential causes of heterogeneity. If the substantial heterogeneity remains significant, and meta-analysis is not appropriate, a narrative summary will be presented.

#### 2.5.9 Publication biases

We will use funnel plot to detect the publication bias if more than ten included studies are available. Furthermore, Egger's regression and Begg's tests will also be applied to detect the funnel plot asymmetry.

#### 2.5.10 Sensitivity analysis

Sensitivity analysis will be conducted to check the robustness of results, as well as the methodological quality, sample size, and the missing data of included studies.

## 3 Discussion

The protocol of this systematic review will be conducted to evaluate the efficacy and safety of cyclophosphamide for the treatment of patients with ALL. Currently, no systematic review and meta-analysis has been performed on this issue. Thus, it is very important and very necessary to run this study to further investigate the efficacy and safety of cyclophosphamide for ALL in both children and adults.

In the current systematic review, we will retrieve all associated literature without language restrictions. All potential studies regarding the cyclophosphamide for ALL will be fully considered to avoid missing any potential trials. The results of this systematic review and meta-analysis will provide a summary of the current evidence on the efficacy and safety of cyclophosphamide for patients with ALL. This evidence may also yield helpful evidence both for clinical practice and the health policy-makers.

## Author contributions

**Conceptualization:** Yue-Rong Zhao, Hong-Mei Song, Lei Ni.

**Data curation:** Yue-Rong Zhao, Hong-Mei Song, Lei Ni.

**Formal analysis:** Yue-Rong Zhao.

**Funding acquisition:** Lei Ni.

**Investigation:** Hong-Mei Song.

**Methodology:** Yue-Rong Zhao, Hong-Mei Song.

**Project administration:** Hong-Mei Song.

**Resources:** Yue-Rong Zhao, Lei Ni.

**Software:** Yue-Rong Zhao, Hong-Mei Song.

**Supervision:** Hong-Mei Song.

**Validation:** Yue-Rong Zhao, Lei Ni.

**Visualization:** Yue-Rong Zhao, Lei Ni.

**Writing – original draft:** Yue-Rong Zhao, Hong-Mei Song, Lei Ni.

**Writing – review & editing:** Yue-Rong Zhao, Hong-Mei Song, Lei Ni.
